# Effectiveness of computer-based stress inoculation training (SIT) counseling approach on anxiety, depression, and stress of students with premenstrual syndrome

**DOI:** 10.1186/s12889-024-18003-0

**Published:** 2024-02-22

**Authors:** Fatemeh Zolfaghary, Hajar Adib-Rad, Fatemeh Nasiri‑Amiri, Mahbobeh Faramarzi, Hajar Pasha, Hemmat gholinia-ahangar

**Affiliations:** 1 Student Research Committee, Master’s student in midwifery counseling, School of Nursing and Midwifery, Babol University of Medical Sciences, Babol, Iran; 2 Social Determinants of Health Research Center, Health Research Institute, Department of Nursing and Midwifery, Babol University of Medical Sciences, Babol, Iran; 3 Population, Family and Spiritual Health Research Center, Health Research Institute, School of Medicine, Babol University of Medical Sciences, Babol, Iran; 4 Clinical Research Development Unite of Rouhani Hospital, Babol University of Medical Sciences, Babol, Iran

**Keywords:** Stress inoculation training, Premenstrual syndrome, Depression, Anxiety, Stress

## Abstract

**Background:**

Premenstrual Syndrome (PMS) is a common public health issue affecting many women of reproductive age worldwide. This study has been designed to investigate of computer-based stress inoculation training (SIT) counseling approach on anxiety, depression, and stress of university students with PMS.

**Methods:**

A randomized trial study with two parallel arms was done from 30 October 2022 to 21 June 2023 on 100 university students aged 18 to 38 at Babol University of Medical Sciences. The participants were randomly divided into two groups intervention and control. The data collection tools included questionnaires on demographic-fertility characteristics, the Premenstrual Symptoms Screening Tool (PSST), the Hospital Anxiety and Depression Scale (HADS), the Perceived Stress Scale (PSS-14), the Sheehan Disability Scale (SDS) and Riff’s Psychological Well-being Scale (RPWS). The data were assessed using chi-square, t-student, ANOVA repeated measure, and linear regression tests. A significance level of *P* < 0.05 was considered for the analysis.

**Results:**

The results of the study showed that the SIT interventions decreased the PMS severity and most psychological factors so in the intervention group, SIT was able to significantly reduce anxiety, depression, perceived stress, and Sheehan’s disability after intervention (*P* < 0.001). Based on multiple linear regression analysis, the most predictors of HADS were the PSS and SDS (β = 0.285, *p* = 0.009 and β = 0.236, *p* = 0.024, respectively).

**Conclusion:**

The computer-based SIT counseling approach could reduce the severity of symptoms and psychological factors in students. Therefore, SIT intervention is recommended to manage their PMS.

**Trial registration:**

IRCT20230130057274N2.

## Introduction

Premenstrual Syndrome (PMS) is a common public health issue among women worldwide in reproductive age [1]. It is one of the most prevalent problems during the reproductive years, which can hurt daily normal life. PMS can interfere with interpersonal relationships. Women with PMS reported a poorer quality of life [2]. This syndrome includes physical and psychological clinical manifestations in the luteal phase of the menstrual cycle, leading to significant distress and functional impairment. Symptoms disappear within a few days [for two days and a maximum of four days] after the onset of menstruation [3, 4]. Women with higher education are more likely to experience PMS symptoms [5] because PMS was shown to significantly affect study-related quality of life. In a study conducted in Iran, PMS was observed in 85 to 90% of women [6], and in another study, its prevalence was reported as 37.8% among students [7].

The symptoms of PMS include abdominal pain, back pain, headaches, bloating, breast tenderness and pain, nausea, constipation, changes in appetite, sleep disturbances, difficulties in concentration, fatigue, joint or muscle pain, weight gain, acne, abdominal bloating, mood swings, anxiety, irritability, nervousness, anger, feelings of losing control, restlessness, mood fluctuations, and crying [3, 8, 9]. Although the etiology of this disorder is uncertain, neurohormones and neurotransmitters, inappropriate inflammatory responses, and oxidative stress are implicated in its development [10]. Because the interaction of the central nervous system with the autonomic, reproductive, and immune nervous systems produces subtle but widespread changes in mood, emotions, sensory processing, appetite, nervous function, etc. In a study conducted on students with PMS, increased feelings of depression, anxiety, fatigue, irritability, pain, and changes in sleep habits were observed. These students sought more support and showed a tendency to utilize healthcare interventions [11].

Girls affected by premenstrual syndrome often face psychological symptoms such as depression [12]. Depression is a significant psychological disorder and a reaction that individuals experience when confronted with distress or a sense of loss [13, 14]. According to the findings of a research study, depression itself exacerbates PMS [15]. Individuals with high levels of anxiety tend to experience higher levels of PMS, and as anxiety levels increase, the severity of PMS also tends to be higher [16]. While PMS is associated with anxiety and depression in women, counseling can not only reduce the intensity of PMS but also alleviate the severity of depression, stress, and anxiety in these individuals [17]. There is a significant relationship between stress levels and the occurrence of PMS [18]. Researchers have reported that stress inoculation training (SIT) is one of the most effective methods for reducing anxiety, depression, and promoting mental well-being [19]. The SIT approach, emphasizing the role of beliefs and cognition in treating psychological problems, is considered a suitable complement to pharmacotherapy and has gained significant attention from practitioners [20].

The coping skills embedded in SIT empower individuals to creatively confront stressful situations and reevaluate their prior beliefs [21]. The results of a study demonstrated a reduction in anxiety, depression, and stress among pregnant women following intervention with virtual and semi-face-to-face SIT techniques [22]. Despite the evidence of premenstrual psychological and physical problems, most research has been reported descriptively. The topic of study is very important, and there are few studies on the effectiveness of one approach in its management. Therefore, it is necessary to solve this gap. Given the significance of the PMS topic in women, the present study aims to investigate the effectiveness of a computer-based SIT counseling approach on anxiety, depression, and stress in students with premenstrual syndrome.

## Materials and methods

### Design and data collection

This randomized trial study with two parallel arms was done on 100 university students aged 18 to 38 [23]at Babol University of Medical Sciences. The participants were divided into two groups: an intervention group and a control group, both of which expressed a willingness to participate in the study. The research was carried out after obtaining approval from the ethics committee of Babol University of Medical Sciences under the ethics code IR.MUBABOL.REC.1401.156, and registered in the IRCT (IRCT20230130057274N2).

**Fig. 1:**
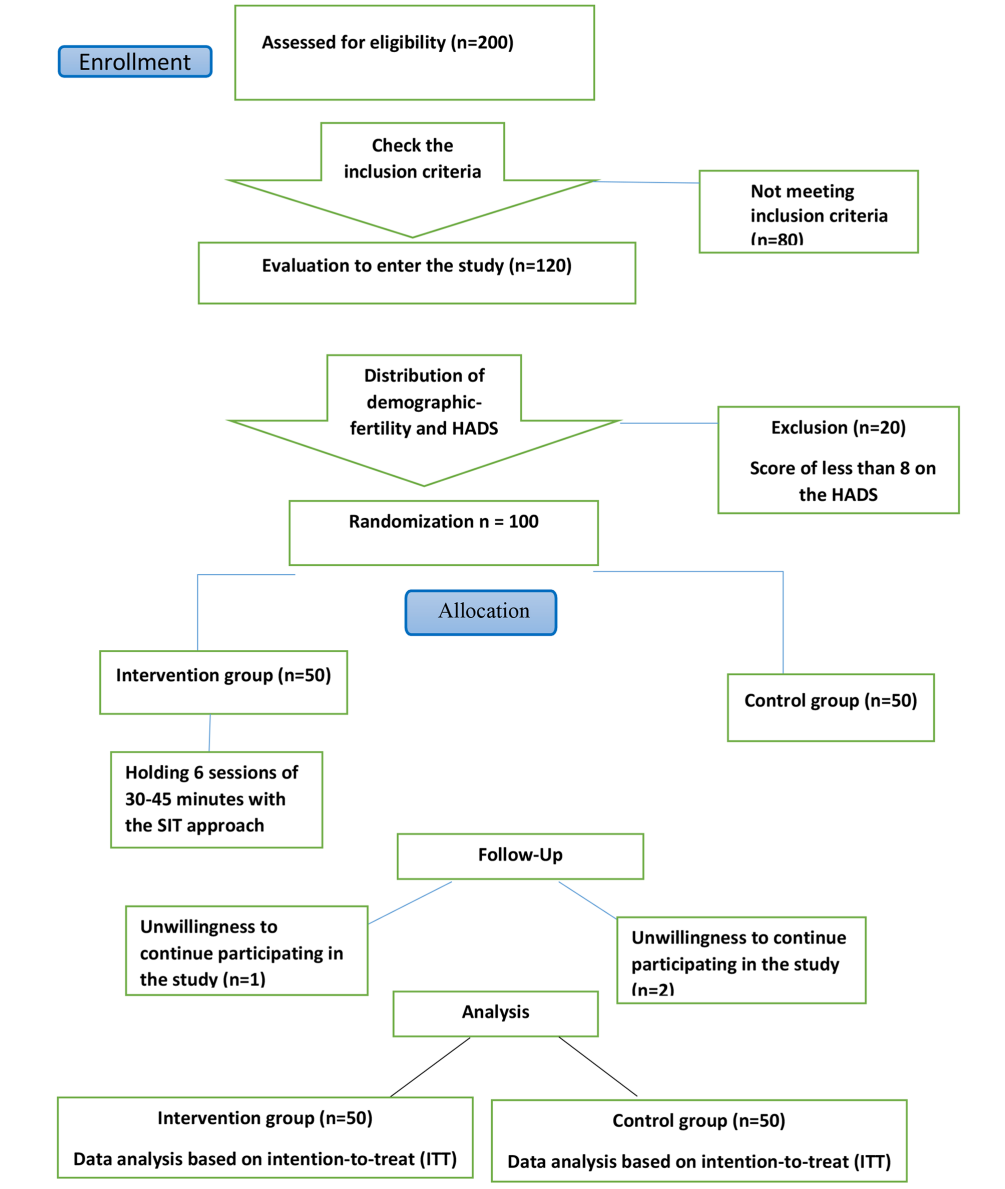
flowchart of participants

The sample size based on the study of Borji-Navan et al. [23] was estimated using the formula with a confidence level of 95% and a power of 80%, resulting in an estimated sample of 90 to account for a possible 10% dropout rate. Therefore, a total of 100 participants were included in the study. Before starting the intervention, out of 200 people who participated in the study, 80 people were excluded from the study due to a lack of inclusion criteria. Therefore, 120 people were evaluated to enter the study and 20 people who had a Score of less than 8 on the HADS were excluded from the study. Therefore, a total of 100 participants were randomly included in the study, with 50 participants in each of the intervention and control groups (Fig. 1).

$$ n=\frac{{\left({Z}_{1-\frac{\alpha }{2}}+{Z}_{1-\beta }\right)}^{2}\left({S}_{1}^{2}+{S}_{2}^{2}\right)}{{\left(d\right)}^{2}}=45$$

$$ \alpha = 0.05\,\beta = 0.20\,{S_1} = 6.99\,{S_2} = 7.14\,\,d = 4.2 $$

After applying the inclusion and exclusion criteria, the students were randomly divided into two groups by random allocation using the alternative block method. The size of the blocks was 4 and using the statistical software, 4 blocks will be produced 25 times. Therefore, a sample list of 100 was generated. Using this randomly generated list, the participants were assigned to two groups of 50 people. The random sequence of people in the intervention or control groups was done through block building with a ratio of 1:1, and the researcher could not predict the placement of the next person in any of the two groups. The randomized list was designed by the methodologist who did not participate in any of the sampling stages of the study. This list was generated by the software and using the sealed envelope site.

The inclusion criteria for the study encompassed being of Iranian nationality, residing in the university dormitories of Babol University of Medical Sciences, completing the written consent form to participate in the study, being diagnosed with PMS (using the premenstrual symptoms screening tool (PSST)), having regular menstrual cycles with intervals of 21 to 35 days and lasting 3 to 7 days for at least the past six months, experiencing 5 symptoms of PMS for two consecutive months (in the time range of 5 days before menstruation and maximum in the first 2 days of menstruation), exhibiting signs of depression or anxiety with a score of 8 or higher, access to the smartphone, and age range from 18 to 38 years. The study’s exclusion criteria consisted of using antidepressant or antianxiety medications during the study, participating in other psychological interventions such as psychotherapy, meditation, and yoga, experiencing stressful events (such as the death of close relatives, severe family conflicts, parental separation, and unfortunate incidents) at least six months before the study, suffering from reproductive system diseases, known chronic diseases, using contraceptive pills, having hormonal disorders and irregular menstruation, currently or historically suffering from severe mental illnesses such as severe depression, bipolar disorder, suicidal thoughts, being addicted to drugs and using psychoactive substances or alcohol, and participants’ unwillingness to continue in the study. The primary outcomes of the study were changes in anxiety, stress and depression scores. The secondary outcomes included changes in Sheehan disability scores, Riff’s psychological well-being, and reduction of PMS symptoms.

### Measurements

The data collection tools included a demographic-fertility characteristics questionnaire, premenstrual symptoms screening tool, and scales of hospital anxiety and depression, perceived stress, Sheehan’s disability, and Riff’s psychological well-being. The main researcher with expertise in midwifery counseling distributed the questionnaires. It should be noted that before starting the work, the main researcher received the necessary training for three months regarding the computerized SIT model under the supervision of a psychology consultant.

#### The demographic- fertility Characteristics questionnaire

used in this study covers various variables, including age, Body Mass Index (BMI), menarche age, duration of bleeding, interval of bleeding, PMS Characteristics (starting time, duration, and intensity), days of absence from class, grade, father’s job, mother’s job, father’s education, mother’s education, residence, satisfaction with income, marital status and family history of PMS.

#### The Premenstrual Symptoms Screening Tool (PSST)

comprises 19 questions divided into two sections. The first section consists of 14 mood, physical, and behavioral symptoms, while the second section assesses the impact of these symptoms on individuals’ lives with 5 questions. Each question is rated on a 4-point scale: none, mild, moderate, and severe, with scores ranging from 0 to 3. The internal consistency of the questionnaire’s items yielded a Cronbach’s alpha of 0.90 for the symptoms section, 0.91 for the impact of symptoms on the life section, and an overall score of 0.93. Additionally, the intra-class correlation between these two sections was 0.80 Moreover, the Content Validity Ratio (CVR) and Content Validity Index (CVI) of the questionnaire were 0.70 and 0.80, respectively [24].

#### The Hospital Anxiety and Depression Scale (HADS)

was used to assess anxiety and depression. This tool simultaneously measures depression and anxiety in outpatients. HADS is a self-reported 14-item instrument. Its reliability, measured using Cronbach’s alpha, is 0.83 for the depression subscale and 0.85 for the anxiety subscale [25]. The questionnaire consists of 4-choice questions and scoring ranges from 0 to 3. Therefore, the scores for the depression and anxiety subscales of the HADS questionnaire fall within the range of 0 to 21, where higher scores indicate higher levels of anxiety and depression. Zigmond and Snaith classify scores for both subscales as follows: scores of 0 to 7 as normal, 8 to 10 as mild, 11 to 14 as moderate, and 15 to 21 as severe [26].

#### The Perceived Stress Scale (PSS-14)

is used to measure general perceived stress over the past month, assessing thoughts and feelings about stressful events, control, overcoming challenges, dealing with psychological pressure, and experiencing stress. The scoring method of the questionnaire is as follows: using a 5-point Likert scale ranging from “never” (score of 0), " rarely” (score of 1), “sometimes” (score of 2), “fairly often” (score of 3), to “very often” (score of 4). Items 4, 5, 6, 7, 9, 10, and 13 are reverse-scored. The lowest score attainable is 0, and the highest score is 56. Hence, the score range is 0 to 56, with higher scores indicating higher levels of stress [27]. Cronbach’s alpha for the PSS-14 has been reported as 0.84, 0.85, and 0.86 in three studies. Additionally, the correlations of this scale with cognitive indicators are also significant, ranging from 0.52 to 0.76 [27].

#### The Sheehan Disability Scale (SDS)

is the most common tool used to measure an individual’s functioning in three domains: academic/occupational, family life responsibilities, and social life. In this scale, the individual assigns a score from 0 to 10 to their functioning in each of the three domains. The scale comprises 10 questions, and for each option, scoring is as follows: 0 (never), 1 to 3 (mild), 4 to 6 (moderate), 7 to 9 (severe), and 10 (very severe) [28].

Sensitivity to change resulting from treatment makes it a suitable and favored tool for clinical specialists to monitor and assess functional disability in the academic, occupational, and social domains [29]. Its reliability has been confirmed in various studies with Cronbach’s alpha ranging from 0.67 to 0.88 [23, 30].

#### Riff’s Psychological Well-being Scale (RPWS)

is a type of self-report tool where respond to a 6-point spectrum from “strongly agree” to “strongly disagree” (ranging from one to six). A higher score indicates better psychological well-being [31]. The short form with 18 questions has been most commonly utilized and applied in various research studies [32]. Results obtained from the assessment of the 18-question version indicated a substantial correlation ranging from 0.70 to 0.89 [33].

### Interventions

At the beginning of the study, all participants completed the 14-item HADS questionnaire. Individuals who scored 8 or higher in either of the subscales, namely depression or anxiety, were identified with symptoms of depression and anxiety and were included in the study. Students who met the inclusion criteria were purposefully selected and then randomly assigned to either the intervention or control group. Subsequently, all questionnaires were administered to the participants within 5 days before menstruation to the first 2 days after menstruation because PMS happens often at this time. Students with moderate to high PMS levels, as determined by the PSST questionnaire, were enrolled in the study. The questionnaires were completed by participants in both intervention and control groups before the intervention, immediately after the intervention, and in the first and second menstrual cycles after the intervention.

In the intervention group, computer-based SIT was individually delivered, consisting of 6 consecutive sessions (one session per week), each lasting 30 to 45 min. The structure of SIT sessions is outlined in Table 1. The participants included students with bachelor’s, master’s, and doctoral degrees.

**Table 1:** Outline of the training sessions

Training sessions	Description of training sessions
Session 1	Conceptualization of the treatment model, gaining awareness of the connection between PMS and negative emotions, acquiring skills for identifying and evaluating thoughts and emotions, relaxation techniques.
Session 2	Developing the ability to identify automatic negative thoughts and initiating their modification, relaxation techniques.
Session 3	Developing the ability to recognize cognitive errors, relaxation techniques.
Session 4	Acquiring skills to correct automatic negative thoughts, relaxation techniques.
Session 5	Developing the ability to assess daily activities, stress coping techniques, relaxation techniques.
Session 6	Designing effective stress coping strategies, addressing challenges in treatment termination, comprehensive relaxation techniques.

The SIT approach leads to improvement in emotional distress and prevents maladaptive psychological responses to stress. The SIT method in the intervention group encompasses a wide range of techniques, including relaxation and breathing exercises, problem conceptualization, identification of negative thoughts, problem-solving and coping skills, concentration and mind distortion techniques, and role-playing [34].

All participants in both intervention and control groups completed the PSST, HADS, SDS, PSS-14, and RPWS questionnaires before the intervention, immediately after the end of the intervention, and the first and the second menstrual cycle after the intervention. The content of the intervention included SIT and PMS. The session outline for the intervention group was as follows: The SIT technique was conducted by an expert psychotherapist (author Dr. M F), who had a license in psychology. Scientific and valid references were used in PMS and psycho-education regarding PMS and its application in SIT [4, 34]. The content of the SIT intervention was scientific and valid [22, 35, 36]. A female assistant (author F Z) had received the necessary training for three months regarding the SIT model under the supervision of a psychology consultant before the trial. After gaining the necessary proficiency in the techniques of this model, he produced all the content inside the computer under the guidance of the psychologist and supervisors (authors Dr. H A-R and Dr. F N-A) and received supervision every week. Computer content with links was sent to students individually. It is necessary to explain that after sending the link during the week, the student had a WhatsApp/phone connection with the subjects for 20–25 min to support them and answered their questions regarding the understanding of the education sessions, as well as how to do the weekly exercises.

It is worth mentioning that no educational intervention was provided to the control group, and they remained on a waiting list. After completing the study and upholding research ethics, participants in the control group were given the option to receive the computer-based SIT intervention, similar to the intervention group, free of charge for 6 sessions.

### Statistical analysis

The data were analyzed using the Statistical Package for the Social Sciences (SPSS) 22.0. software. Descriptive statistics, such as mean, standard deviation, frequency distribution table, and relative frequency, were calculated. In addition, ANOVA repeated measure and linear regression analysis were used for data analysis. The significance level of the tests was considered to be *P* < 0.05.

## Results

In this study, 100 people (50 people in the intervention group and 50 people in the control group) were evaluated. The results of this study showed that the mean age of the participants in the intervention group was 25.96 (4.93) years, and in the control group was 25.07 (4.77) years. The average body mass index of the participants in the intervention group was 23.56 (3.71), and in the control group was 22.83 (3.46). In addition, the percentage of family history of PMS was 26 (56.5) in the intervention group and 25 (55.6) in the control group (Table 2).

**Table 2:** Demographic- fertility characteristics of students with premenstrual syndrome (*n* = 100)

**Variables**	**Intervention group (***n* **= 50)**	**Control group (***n* **= 50)**	**Total (***n* **= 100)**	***P- value*** ^†^
Age (mean ± SD, year)	25.96 (4.93)	25.07 (4.77)	25.52 (4.8)	0.384
BMI* (kg/m^2^ )	23.56 (3.71)	22.83 (3.46)	23.20 (3.5)	0.334
Menarche age (year)	12.93 (1.54)	12.69 (1.18)	12.81 (1.37)	0.396
Duration of bleeding (day)	6.26 (1.46)	5.71 (1.27)	5.99 (1.39)	0.060
Interval of bleeding (day)	28.48 (2.38)	28.62 (2.188)	28.55 (2.27)	0.765
PMS time (day)	4.57 (2.51)	3.93 (3.11)	4.25 (2.92)	0.305
Days of absence from class	1.67 (2.47)	1.62 (2.14)	1.65 (2.34)	0.917
Onset of symptoms (year)	11.98 (1.86)	16.67 (4.44)	14.30 (4.11)	0.001>
**Variables**	**Intervention group** **f (%)**	**Control group** **f (%)**	**Total** **f (%)**	**P- value** ^**††**^
**Grade**				0.344
BSc	19 (41.3)	25 (55.6)	44 (48.4)	
MSc	22 (47.8)	15 (33.3)	37 (40.6)	
PhD	5 (10.9)	5 (11.1)	10 (11.0)	
**Father’s job**				0.374
Employee	10 (21.7)	14 (31.1)	24 (26.4)	
Worker	23 (50.0)	17 (37.8)	40 (44.0)	
Retired	13 (28.3)	14 (31.1)	27 (29.6)	
**Mother’s job**				0.452
Housewife	34 (78.2)	30 (66.7)	60 (72.5)	
Employee	10 (21.7)	15 (33.3)	25 (27.5)	
**Father’s education**				0.751
High school	10 (21.7)	9 (20.0)	19 (20.9)	
Diploma	13 (28.3)	15 (33.3)	28 (30.8)	
University	22 (47.8)	21 (46.7)	43 (47.3)	
**Mother’s education**				0.786
High school	11 (23.9)	10 (22.2)	21 (23.1)	
Diploma	16 (34.8)	16 (35.6)	32 (35.2)	
University	18 (39.1)	19 (42.2)	37 (40.7)	
**Residence**				0.197
Rural	3 (6.5)	7 (15.6)	10 (11.0)	
Urban	43 (93.5)	38 (84.4)	81 (89.0)	
**Satisfaction with income**				0.641
Satisfied	6 (13.0)	7 (15.6)	13 (14.3)	
Relatively satisfied	26 (56.5)	21 (46.7)	47 (51.6)	
Un satisfied	14 (30.4)	17 (37.8)	31 (34.1)	
**Marital status**				0.106
Single	33 (71.7)	39 (86.7)	72 (79.1)	
Married	10 (21.7)	6 (13.3)	16 (17.6)	
Divorced	3 (6.5)	0 (0.0)	3 (3.3)	
Family history of PMS**				0.926
Yes	26 (56.5)	25 (55.6)	51 (56.0)	
No	20 (43.5)	20 (44.4)	40 (44.0)	

^*^Body mass index

**Premenstrual syndrome

^**†**^The data were assessed using t-test

^**††**^The data were assessed using chi-square test

**Fig. 2:**
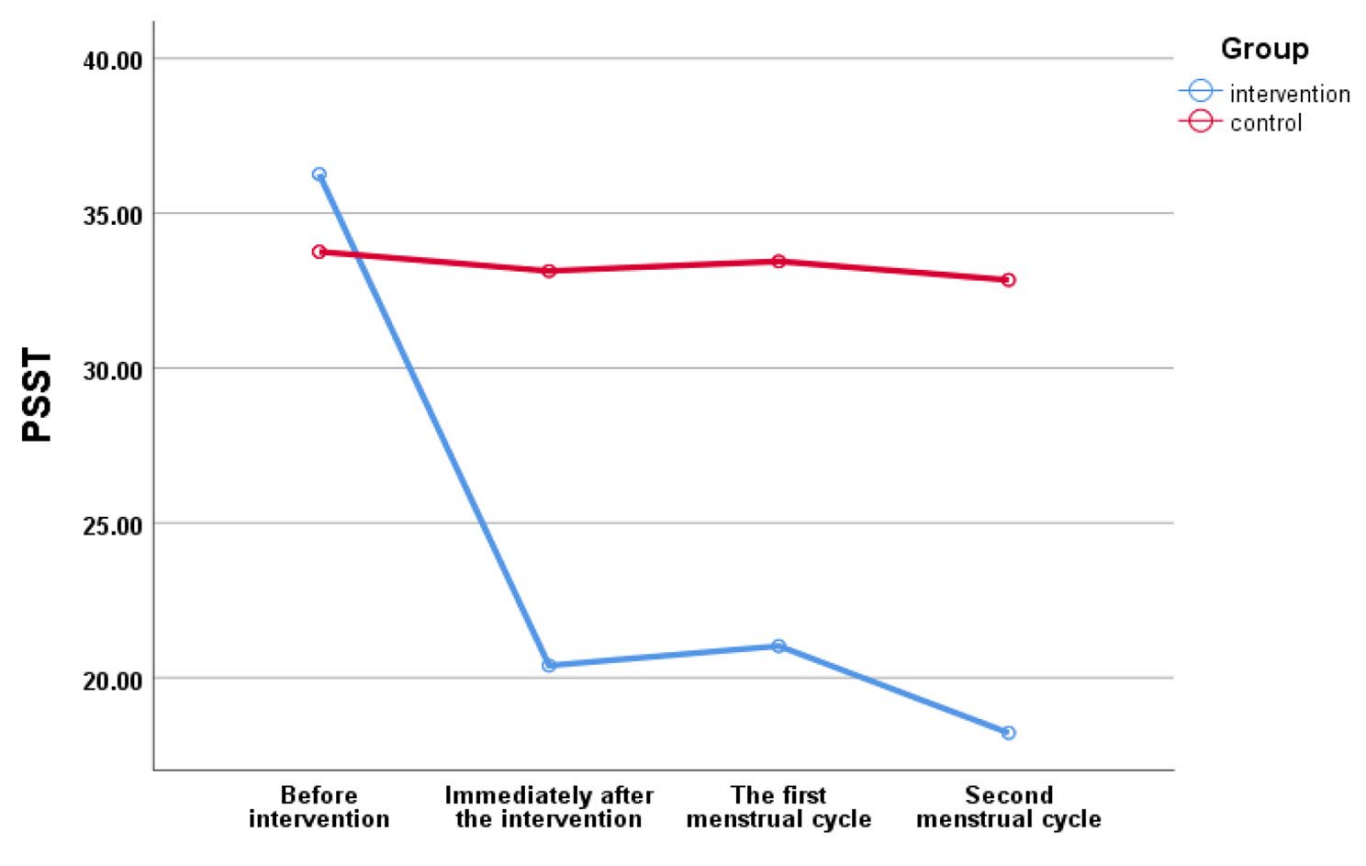
Status of premenstrual symptoms screening tools in students with premenstrual syndrome (*n* = 100)

**Fig. 3:**
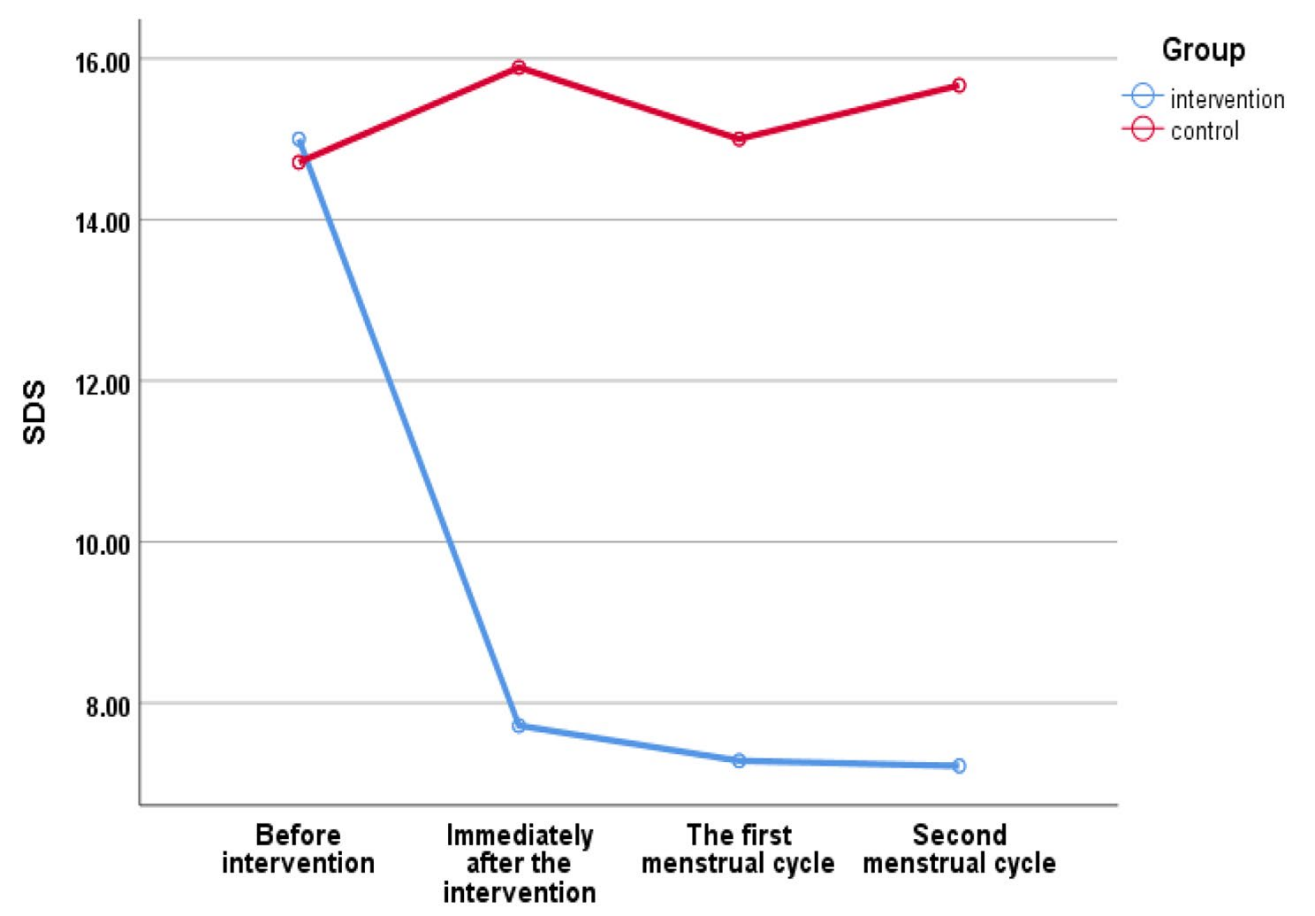
Status of Sheehan disability scale in students with premenstrual syndrome (*n* = 100)

Based on the results presented in Table 3, the mean scores of PSST, HADS, PSS-14, and SDS in the intervention and control groups before the intervention were statistically not significant, while there was a significant difference in the immediately after the intervention, the first and second menstrual cycles after the intervention in the two groups. These results showed the efficiency and positive impact of the SIT approach. There was no significant difference between the two groups in the mean scores of RPWS in before and after the intervention. However, there was a difference in the mean between and within the groups with a weak effect size in RPWS Table 3. Also, according to Fig. 2, the mean PSST score before the intervention in the intervention group was 36.2, but immediately after the intervention, it reached 18.2, which was a significant difference (*P* < 0.05). According to Fig. 3, the mean SDS score in the intervention group before the intervention was 15, but it reached 7.2 immediately after the intervention, which had a significant difference (*P* < 0.05).

**Table 3:** Group comparisons in the status of PSST, HADS, PSS-14, SDS, and RPWS before and after intervention (*n* = 100)

Measures study group (*n* = 100)	Intervention, Mean (SD)					Within- group				Between- group			
	Mean (SD) T0^†^	Mean (SD) T1^††^	Mean (SD) T2^†††^	Mean (SD) T3^††††^	P- Value†	Mean diff (CI 95%) T0-T1	Mean diff (CI 95%) T0-T2	Mean diff (CI 95%) T0-T3	Eta effect size	Mean diff (CI 95%) T0-T1	Mean diff (CI 95%) T0-T2	Mean diff (CI 95%) T0-T3	Eta effect size
**Depression** Intervention Control	**10.89 (2.93)** **10.66 (3.11)**	**5.65 (2.45)** **11.20 (1.57)**	**6.02 (3.19)** **11.04 (1.22)**	**6.21 (3.07)** **10.91 (1.47)**	< 0.001	**5.23 (4.20_6.27)** **− 0.533 (-1.31_.252)**	**4.86 (3.52_6.21)** **− 0.377 (-1.36_.606)**	**4.67 (3.19_6.15)** **− 0.244 (-1.13_.648)**	**0.19**	**5.77 (4.48_7.06)**	**5.24 (3.59_6.89)**	**4.91 (3.20_6.63)**	**0.62**
**Anxiety** Intervention Control	**11.39 (2.54)** **10.84 (2.45)**	**6.45 (2.20)** **11.24 (1.43)**	**6.73 (2.64)** **11.33 (1.47)**	**6.65 (2.79)** **11.48 (2.01)**	< 0.001	**4.93 (4.02_5.84)** **− 0.400 (-1.17_.375)**	**4.65 (3.69_5.60)** **− 0.488 (-1.28_.302)**	**4.73 (3.62_5.84)** **− 0.644 (-1.54_.257)**	**0.23**	**5.33 (4.15_6.51)**	**5.14 (3.91_6.36)**	**5.38 (3.96_6.79)**	**0.59**
PSST* Intervention Control	**36.26 (7.65)** **33.75 (6.40)**	**20.3 (6.79)** **33.13 (5.3)**	**21.02 (7.20)** **33.44 (5.48)**	**18.21 (6.15)** **32.84 (5.72)**	< 0.001	**15.86 (12.98_18.75)** **0.622 (-1.08_2.32)**	**15.23 (12.62_17.85)** **0.311 (-1.47_2.09)**	**18.04 (15.60_20.48)** **0.911 (-1.21_3.03)**	**0.42**	**15.24 (11.92_18.57)**	**14.92 (11.78_18.06)**	**17.13 (13.93_20.32)**	**0.51**
PSS-14** Intervention Control	**29.02 (7.89)** **27.33 (5.68)**	**24.69 (5.54)** **27.35 (2.83)**	**25.04 (5.59)** **28.02 (2.64)**	**25.8 (4.97)** **28.22 (3.13)**	< 0.001	**4.32 (1.71_6.93)** **− 0.022 (-1.70_1.65)**	**3.97 (1.47_6.47)** **− 0.689 (-2.48_1.10)**	**3.15 (.519_5.78)** **− 0.888 (-2.73_0.953)**	**0.04**	**4.34 (1.27_7.42)**	**4.66 (1.62_7.71)**	**4.04 (.858_7.22)**	**0.05**
SDS*** Intervention Control	**15.0 (5.46)** **14.71 (4.83)**	**7.71 (3.28)** **15.88 (3.41)**	**7.28 (3.24)** **15.0 (3.04)**	**7.21 (3.94)** **15.66 (3.69)**	< 0.001	**7.28 (5.82_8.74)** **-1.17 (-2.17_-0.182)**	**7.71 (6.11_9.31)** **− 0.289 (-1.49_.913)**	**7.78 (5.98_9.57)** **− 0.956 (-2.09_0.181)**	**0.27**	**8.46 (6.71_10.20)**	**8.00 (6.02_9.98)**	**8.73 (6.63_10.84)**	**0.49**
RPWS**** Intervention Control	**67.76 (7.16)** **68.57 (6.99)**	**70.71 (5.74)** **68.84 (7.04)**	**70.28 (5.23)** **69.53 (6.84)**	**70.13 (6.93)** **70.44 (6.46)**	**0.152**	**-2.95 (-4.94_-0.967)** **− 0.266 (-1.54_1.01)**	**-2.52 (-4.82_-0.220)** **− 0.955(-2.31_.4001)**	**-2.36 (-4.86_-0.126)** **-1.86 (-3.33_-0.398)**	**0.05**	**-2.68 (-5.03_-0.346)**	**-1.56 (-4.21_1.08)**	**− 0.502 (-3.37_2.36)**	**0.01**

*Premenstrual symptoms screening tools

**Perceived Stress Scale

***Sheehan Disability Scale

****Riff’s Psychological well-being scale

^**†**^ Before intervention

^**††**^ Immediately after the intervention

^**†††**^ First menstrual cycle

^**††††**^ Second menstrual cycle

To perform multiple linear regression, first, we listed the results of simple linear regression in Table 4 and considered the significance level to be 0.2 in this part. Then, we entered the variables that were significant into the model. Table 5 shows the results of multiple regression that the final model fitted at a significance level of 0.05.

**Table 4:** Related factors with HADS in simple linear regression analysis in students with premenstrual syndrome (*n* = 100)

Variables	Β^*^	95% CI^**^		P- value^†^
		Lower Bound	Upper Bound	
**Constant**				0.000
BMI***	0.022	− 0.242	0.297	0.839
**Residence**	− 0.031	-3.527	2.631	0.773
**Marital status**	0.150	− 0.533	3.282	0.156
PMS time****	0.002	− 0.233	0.238	0.984
**Mother’s education**	− 0.064	-1.563	0.837	0.549
**Satisfaction with income**	0.071	− 0.952	1.931	0.502
Family history of PMS****	− 0.145	-3.261	0.580	0.169
PSS-14*****	0.394	0.133	0.391	0.000
RPWS******	− 0.165	− 0.243	0.028	0.117
SDS*******	0.340	0.127	0.482	0.001

^*^Standardized coefficients

** Confidence interval

***Body mass index

****Premenstrual syndrome

*****Perceived Stress Scale

******Riff’s Psychological well-being scale

*******Sheehan Disability Scale

^**†**^ The data was assessed using simple linear regression

**Table 5:** Related factors with HADS in multiple linear regression analysis in students with premenstrual syndrome (*n* = 100**)**

Variables	Β^*^	95% CI^**^		P- value^†^
		Lower Bound	Upper Bound	
**Constant**		6.67	28.23	0.002
**Marital status**	0.026	-1.62	2.09	0.802
Family history of PMS***	0.102-	-2.74	0.860	0.302
RPWS****	− 0.067	− 0.171	0.084	0.498
PSS-14*****	0.285	0.029	0.394	0.009
SDS******	0.236	0.035	0.392	0.024

^*^Standardized coefficients

** Confidence interval

***Premenstrual syndrome

**** Riff’s Psychological well-being scale

*****Perceived Stress Scale

******Sheehan Disability Scale

^**†**^The data was assessed using multiple linear regression

Factors related to HADS in simple linear regression analysis are presented in Table 4. Factors related to HADS in multiple linear regression analysis are presented in Table 5. The most predictors of HADS were the PSS (β = 0.285, *p* = 0.009) and SDS (β = 0.236, *p* = 0.024). After adjusting for other variables, the multiple regression analysis indicated that PSS and SDS were significantly associated with HADS. Indeed, the study results revealed that as the level of stress and disability in individuals with PMS increases, their level of anxiety and depression will also be higher.

## Discussion

The results of this study demonstrated that in the intervention group, the mean score of PSST significantly decreased after the intervention compared to before. This reduction was observed immediately after the intervention and in the first and second menstrual cycles after the intervention, and it was statistically significant. In line with our study, the results of one study aimed at the effects of curcumin on premenstrual syndrome showed that in women with PMS, the mean PMS score was significantly lower at the end of the study [37]. The study by Ahmadi et al., with the aim of the effect of Zinc supplementation on the improvement of PMS showed that after a 24-week intervention, physical and psychological symptoms of PMS, such as irritability, anxiety, mood swings, over-eating, breast tenderness, headache, muscle pain, bloating, and weight gain, significantly decreased in the intervention group compared to the placebo group (*P* < 0.001) [38]. In another study, with the aim of the effectiveness of a school-based health education program to improve the symptoms of PMS in high school girls, PSST was also used to diagnose PMS or premenstrual dysphoric disorder (PMDD). The results indicated a significant difference in the proportion of individuals with moderate and severe PMS and PMDD between the intervention and control groups (*P* < 0.001), but these proportions did not differ significantly between the two groups at the beginning of the study [39]. The use of cognitive-behavioral therapy methods can be effective in reducing premenstrual syndrome symptoms, but their effectiveness is more prominent immediately after the intervention period compared to other time intervals. Increasing the time after the intervention can cause the person to forget to perform these methods, so it will be more effective if it is done continuously. Therefore, researchers need to adopt a solution for the continuous efficacy of these methods, allowing individuals to utilize them in any time interval to reduce the severity of PMS symptoms.

The results of this study indicated that the average HADS score of students with PMS significantly decreased after the intervention compared to before. The mean scores of anxiety and depression before the intervention significantly decreased immediately after the intervention and in the first and second menstrual cycles after the intervention. In one study, with the aim of the effect of smartphone psychotherapy on fear reduction after a 24-week intervention, the mean scores of anxiety and depression in the participants of the intervention group showed a significant difference. These scores did not show a statistically significant difference between week 24 and week 8, although the depression score had significantly decreased in week 24 (*P* = 0.03) [40]. Another study to apply pressure to the LIV3 and LI4 on the symptoms of premenstrual syndrome demonstrated that the number of individuals with moderate/severe PMS in the intervention group decreased in the second and third cycles compared to the placebo group (*P* < 0.04). Additionally, the scores of depression and anxiety in the intervention group significantly decreased in the second and third cycles compared to the placebo group (*P* < 0.05) [41]. The study by Vicariotto et al. with the aim of the effectiveness dietary supplement of vitamin B6 with four herbal extracts for sleep disturbances and anxiety associated with the menstrual cycle showed a 23.2% improvement in the scores of anxiety and depression in the intervention group [42]. In the mentioned studies, researchers showed a greater tendency to use pharmacological treatment to reduce anxiety and depression related to PMS. However, our study had psychological and counseling aspects, and both approaches have been effective in achieving the goal of reducing significant anxiety and depression in individuals with PMS. Considering the reluctance of many individuals to use pharmacological treatment, internet-based counseling methods can provide a more effective solution for reducing the severity of PMS symptoms. Taking help from psychotherapy leads to reducing the use of drugs and reducing their side effects. Therefore, mood swings will no longer surprise people, and they can take more steps to self-care.

Another finding of this study was the reduction in PSS-14 scores in the participants of the intervention group compared to the control group in after the intervention. In a study conducted by Mohebbi et al., with the aim of the comparison between the lifestyles of university students with and without PMS, it was determined that dietary style (*p* = 0.001) and perceived stress (*p* = 0.001) were different between two groups of dormitory students with and without PMS [43]. In another study among medical interns, a significant relationship between perceived stress and PMS was observed [44]. From the mentioned studies, it can be inferred that the living conditions of students can play a role in creating stress or increasing its intensity. Therefore, counseling should be taken along with medical methods to reduce stress in individuals with PMS, and health policymakers should pay special attention to this issue. Stress can make depression symptoms worse. Therefore, we should advise people to get enough sleep and rest. Relaxation and muscle relaxation exercises, massage, or deep breathing are useful to help reduce insomnia.

Based on the results of this study, the mean SDS scores, which encompass academic/occupational, family life responsibilities, and social life domains, showed a significant difference after the intervention compared to before. The mean SDS score in the intervention group had a significant reduction compared to the control group in three time intervals after the intervention (immediately after the intervention, the first menstrual cycle after the intervention, and the second menstrual cycle). A study in line with our research with the aim of internet-based cognitive-behavioral therapy for premenstrual syndrome demonstrated that the mean score severity of PMS in the intervention group was significantly lower than the control group after the intervention (*P* < 0.001). Additionally, this study indicated a significant difference in the mean SDS score related to PMS after the intervention compared to before (*P* < 0.001) [45]. Another study to evaluate PMS and fibromyalgia—Similarities and common features showed that patients with PMDD had higher levels of sensitivity, higher psychological symptoms, greater physical disability, and lower quality of life [46]. Considering the results of the mentioned studies, the role of individuals in family and social life and the vulnerability of women during the menstrual period must also be considered in reducing the severity of symptoms related to PMS. PMS causes a person’s inability to perform daily activities and social interactions, which reduces work efficiency. Researchers should investigate these aspects to achieve more comprehensive and complete results.

According to the results of this study, there was no significant difference in the mean RPWS of students after the intervention compared to before, and it was not statistically significant. One study in line with our research with the aim of the effect of mindfulness-based stress reduction training on emotion regulation difficulties and psychological well-being in PMS showed that mindfulness-based stress reduction could be a useful intervention method for increasing psychological well-being and reducing emotion regulation problems in women with PMS [47]. Therefore, due to the non-conformity of our study results with the mentioned studies, other interventions should be designed to promote menstrual health and reduce symptoms in women with this syndrome. In general, various factors such as stress, anxiety, depression, economic issues, living conditions, physical symptoms, and psychological issues affect PMS. This syndrome has widespread consequences, affecting physical health. The level of social, occupational, and educational activities, individuals’ efficacy, and family relationships are significantly related to PMS, as it has both psychological and physical aspects [1]. Thus, empowering girls and women through increased education for reducing PMS symptoms is essential.

The results of this study demonstrated that there was no significant correlation between anxiety and depression in students with any of the demographic characteristics, but there was a significant correlation between anxiety and depression in students with PSS-14 and SDS. The study of Negi et al. with the aim of the effect of Menstrual abnormalities and their association with lifestyle patterns in adolescent girls indicated that PMS has a direct relationship with nutrition, diet, and physical activities, which also affect the body mass index of girls [48]. The study by Hooshiar et al. with the aim of the effect of a modified alternate-day fasting diet on the severity of PMS and health-related quality of life in women overweight or obese concluded in their study that body fat quantity has a role in menstrual and ovulatory cycles and can lead to menstrual problems. The primary treatment for weight reduction in obese patients includes caloric restriction and weight loss [49]. Obese women usually have a sedentary lifestyle. They are at risk of stress, depression, and lack of sleep and exercise less, which may be one of the reasons for the prevalence of PMS in them. The results of another study with the aim of the associated factors with PMS and PMDD among female medical students showed that premenstrual physical and psychological symptoms can lead to significant consequences for adolescents and young women, affecting their relationships, daily functioning, and quality of life [50]. In another study, it was reported that obesity, a sedentary lifestyle, consumption of high-sodium, high-fat, high-sugar diets, carbonated beverages, and low consumption of fruits and vegetables were associated with a higher prevalence of PMS [51]. The study by Abdolrahmi et al. with the aim of the effect of Fordyce happiness training on marital satisfaction and mental health in women with PMS, demonstrated that PMS has various physical and psychological effects. Since healthy family relationships and normal marital interactions are affected by women’s physical and psychological health, any disruption in this area reduces marital satisfaction and consequently endangers mental health and family survival. Therefore, marital status and having healthy marital relationships can significantly impact the severity of PMS symptoms [52]. Another study, with the aim of the effect of PMS on the quality of work life in working women in South India, showed the highest level of education, occupation, and sexual activity were significantly related to PMS and work-related quality of life in women with PMS. A high prevalence of PMS was present in working women, significantly affecting their work-related quality of life [53]. Considering the lack of conformity between our study results and other studies, it cannot be definitively concluded whether obesity, overweight, or marital status are related to the intensity of PMS symptoms, or if there are still gaps that require further extensive research.

In general, several factors such as stress, anxiety, depression, economic issues, living conditions, physical symptoms, and psychological issues are effective in PMS, and this syndrome has wide consequences. PMS has a great impact on women’s physical health. The amount of social activities, work, education, people’s efficiency, and family relationships are significantly related to this syndrome because it has both psychological and physical aspects. Therefore, it is necessary to empower girls and women by promoting more education to reduce PMS symptoms.

### Limitations and strengths

The limitations of our study include conducting the study only on dormitory students, which could restrict the generalizability of the results to other individuals. Another limitation is the follow-up duration, as participants were followed up to 2 months after the intervention. If this time interval were longer, the results might have been more varied. Also, another limitation was that only a questionnaire was used to diagnose depression and anxiety. It is recommended that clinical interviews be used for diagnosis in future studies. Among the strengths of the current study, the use of virtual and internet-based training can be highlighted, which was an effective choice considering the digital age today and the advantages of time and cost savings. Another strength was the presence of the primary researcher in the dormitory, and close communication with participants, addressing their ambiguities, and the utmost cooperation and responsiveness to actively involve them in the study through effective questionnaires. It should be noted that all the questionnaires were completed by the participants. In addition, considering the presence of women in society, their active role as students, and the importance of their health in physical and mental dimensions, computer SIT training can have an effect in reducing the severity of PMS in students and improving their ability and self-efficacy.

## Conclusion

Overall, the results of our study showed that the computer-based SIT approach was able to effectively improve adverse psychological outcomes in students. Therefore, this method can be used along with other treatment methods to reduce the severity of PMS symptoms in women. However, policymakers in the health sector, recognizing the active presence of women in society and education, should adopt new, efficient, and cost-effective interventions to empower women and substantially improve PMS symptoms. Therefore, health policymakers can be suggested to use the findings of this study to manage PMS.

## Data Availability

All data generated and analyzed during this study are included in this article. Datasets this study are available from the corresponding author upon reasonable request.

## Abbreviations

PMS: Premenstrual syndrome
BMI: Body mass index
PSST: Premenstrual symptoms screening tools
HADS: Hospital anxiety and depression scale
PSS: Perceived Stress Scale
SDS: Sheehan Disability Scale
RPWS: Riff’s psychological well-being scale
SPSS: Statistical product service solution
CI: Confidence interval
